# The Responsibility of Silence

**Published:** 1909-10-23

**Authors:** 


### EDITOR'S LETTER-BOX.

### "THE RESPONSIBILITY OF SILENCE."

*To the Editor of* THE HOSPITAL.

SIR,—Your leading article on the above subject has, I must confess, hit me, personally, rather hard. I have, to my shame, before now held my peace when the right course would have been to speak. At the same time there is this much to be urged in excuse, that my future career might have been—almost certainly would have been—much injured had I done what I knew it was my duty to do; and in this imperfect world no man can afford to neglect the consideration of how he is going to make his living.

May I trespass on your space long enough to illustrate my meaning from my own experiences? When I was a house surgeon I was present at an operation conducted by a gentleman whose methods are those of a prosector in anatomy rather than a surgeon. His procrastination and the time he contrives to spend over the simplest operations are unbelievable except to those who have assisted him or anæsthetised for him. I assure you that he quite commonly requires an hour and three-quarters to two hours to perform an ordinary complete amputation of the breast, and I have known him occupy two and a half hours over a simple uncomplicated ovarian cyst. Well, this operation was both tedious and severe, and the patient was an old man of impaired constitution. After an hour or so the anæsthetist found it necessary to do artificial respiration for a short time, at the same time warning the operator to finish his task quickly. After another half hour the same necessity arose, and the warning was repeated. The surgeon ignored his anæsthetist completely, and the patient a few minutes later died on the table. Now here was a very gross case, in which the anæsthetist and I took the responsibility of silence. I admit we both acted wrongly; but I ask you to consider what would have happened had we told the whole truth. First, we should probably not have been believed, for the surgeon lives in the region of Cavendish Square. Next, even if we had been, we should most likely have had to resign our posts; at least, either we should or else the surgeon, and I shrewdly suspect which it would have been. To me this would have meant little at the time, but to the anæsthetist it would have been a serious matter. Lastly, I should never have been able to stand for election to the visiting staff, on which, I may say, the surgeon in question and myself are now colleagues! I don't defend in the least the transaction recorded above; it was grossly immoral from start to finish on my part. But I do point out that this man's incapacity is well known to the other surgeons, and that if they will do nothing in the matter it is rather difficult for a house surgeon to make the first move.

Let me now recount yet another difficult position. When I was a house physician a certain new form of treatment for a certain terrible disease was under trial. This treatment has long since been pronounced on all hands a complete failure. The treatment was not only not beneficial, but actually harmful, and caused also a good deal of pain. At the time I speak of this was not generally known in the profession, though every house officer without exception became convinced of it after a few weeks' experience. After a short trial it was abandoned and condemned by all the visiting staff of the hospital except one. This latter found it convenient to publish papers in the recognised medical press, saying that the remedy certainly appeared to do good in certain cases, though it was too early to pronounce definitely upon it; and on the strength of these papers he made a great deal of money in private practice. It may be added that it was not by any means through want of clinical acumen that he failed to arrive at the conclusions reached by everyone else on the subject of this remedy. At this juncture he one day sent up from another hospital a child about ten years old, with a line commanding her admission and the commencement of this particular treatment. Convinced that the treatment would be both prejudicial and painful, I took advantage of the fact that all my beds were occupied to send the patient back to the special hospital whence she had been brought, a hospital at which her opportunities of recovery were far greater than any she could enjoy elsewhere. The upshot was a stormy interview with the consultant in question, who expressed his sentiments towards me in most pointed language. Another result was that the child was actually saved from the treatment in question. On this occasion, then, I did my duty, or part of it at any rate. But in consequence I incurred the resentment of a personage whose power to influence my future prospects was very considerable indeed.

One more actual experience and I have done. Within my knowledge the senior physician of a hospital, a man of great talents—almost of genius—in his younger days, was in such an impaired state of mind that he was directly responsible for the deaths of several patients. As he was within a year or two of retirement under the hospital rules, no attempt to accelerate his superannuation was made by his colleagues, who, of course, desired to avoid an open scandal. Now in such circumstances I hold very strongly that those colleagues committed a most grave dereliction of public duty. They may not have known the whole truth, as the house physicians knew it, but they knew quite enough to justify them in acting. What, then, is a house physician to do when he finds himself accessory to such a miscarriage of justice? I should be very loth to apply to him the condemnation due to the visiting staff, though from a strictly ethical standpoint I suppose his delinquency would be just as great.

I fear I have written at unconscionable length, but as one whose personal experience of the problems you set forth is perhaps unusually large, I have felt that that experience may be useful in illustrating the complexity of the subject. I trust that, after the plain speaking I have indulged in, you will not object to withholding my name from publication.

I am, Sir, yours faithfully,

London, October 16, 1909. M. D.

### LEAGUE OF MERCY.

With the sanction of H.R.H. the Prince of Wales, Grand President of the League of Mercy, grants amounting to £1,980 have been made to extra metropolitan hospitals. The total sum to date handed over by the League to the King Edward's Hospital Fund for London is now £116,000, in addition to which the sum of £5,476 has been given to extra-metropolitan hospitals of counties collecting for the League.

